# Stroke Outcome and Determinants among Patients with and without Diabetes in a Tertiary Hospital in Ghana 

**DOI:** 10.1155/2018/7521351

**Published:** 2018-09-12

**Authors:** Josephine Akpalu, Alfred E. Yawson, Foster Osei-Poku, Yacoba Atiase, Ernest Yorke, Patrick Adjei, Kodwo Nkromah, Albert Akpalu

**Affiliations:** ^1^Department of Medicine and Therapeutics, School of Medicine and Dentistry, College of Health Sciences, University of Ghana, P.O. Box GP 4236, Accra, Ghana; ^2^Department of Biostatistics, School of Public Health, College of Health Sciences, University of Ghana, P.O. Box LG 13, Legon, Accra, Ghana; ^3^Department of Medicine and Therapeutics, Korle Bu Teaching Hospital, P.O. Box KB 77, Korle Bu, Accra, Ghana

## Abstract

**Background:**

Diabetes mellitus, a well-established independent risk factor for stroke, has varied association with stroke outcome from previous studies. This study investigated stroke outcome and determinants among patients with and without diabetes in a tertiary hospital in Ghana.

**Methods:**

A prospective study conducted among stroke patients with and without diabetes admitted in a Ghanaian tertiary hospital. Baseline clinical and biochemical data were documented. Functional stroke outcome was evaluated at 1, 3, and 6 months after stroke using the modified Rankin Scale.

**Results:**

Number of participants enrolled were 326 and 105 (32.20%) had diabetes. Higher proportions of diabetes patients had poor functional stroke outcome at 1, 3, and 6 months (79%, 75.23%, 73.33%) compared with those without diabetes (70.13%, 65.16, 61.99) (p>0.05). Stroke patients with diabetes had lower survival compared with those without diabetes (p=0.0745). Mortality at 6 months was more likely among ischaemic stroke patients with diabetes compared with those without diabetes (Odds Ratio 2.037; CI: 1.058-3.923). Determinants of poor functional stroke outcome for diabetes patients were older age (Adjusted Odds Ratio (AOR)-1.07; CI-1.03-1.12), female gender (AOR-3.74; CI-1.26-12.65), and pneumonia (AOR-11.32; CI-1.93-220.05) whereas the determinants for those without diabetes were unemployment (AOR-4.19; CI-1.24-19.50), speech abnormalities (AOR-1.99; CI1.08-3.73), and pneumonia (AOR-4.05; CI-1.83-9.77). High fasting plasma glucose (HR-1.15; CI-1.07-1.23), elevated temperature (HR-1.41; CI-1.11-1.79), and pneumonia (HR-2.25; CI-1.44-3.50) were determinants of low survival among all stroke patients.

**Conclusion:**

Trends towards poorer functional outcome and reduced survival were found among Ghanaian stroke patients with diabetes compared with those without diabetes. Older age, female gender, pneumonia, elevated temperature, and fasting plasma glucose were determinants of adverse outcome in stroke patients with diabetes.

## 1. Introduction

Diabetes mellitus (DM) is a well-known independent risk factor for stroke and its worldwide prevalence has been estimated to rise with Sub-Saharan Africa experiencing the highest increase [1–3]. Stroke is one of the most common causes of death and long-term disability [4, 5] and several studies have suggested that DM is associated with a higher mortality from stroke [6–8].

The prevalence of DM among stroke patients ranges between 21 and 44% [6, 7, 9]. A number of studies have shown that DM is a predictive factor for dependency [7, 10] and recurrent stroke [8, 11] and has also been associated with an increase in stroke mortality [6–8]. On the other hand, other studies have found no such association between diabetes and poor stroke outcome [12, 13].

Although stroke has been reported to be an important cause of death and is increasingly becoming a major health problem in some parts of Africa, comprehensive stroke surveillance data for Africa are limited [14, 15]. In Ghana, data on the prevalence of DM among stroke patients and its impact on patient outcomes are lacking. This study investigated the post-discharge functional outcome trends and the associated factors among stroke patients with and without comorbid diabetes in a tertiary hospital in Ghana.

## 2. Methods

This was a prospective study undertaken at the stroke unit of the Korle Bu Teaching Hospital, the main tertiary hospital for southern Ghana. The stroke unit provides both in- and outpatient services to stroke patients referred to the hospital.

Stroke patients admitted to the stroke unit with the diagnosis confirmed by either a Computed Tomography Scan (CT Scan) or Magnetic Resonance Imaging (MRI) and from whom informed consent was obtained were eligible for enrolment. Individuals who did not fulfil the above criteria were excluded. Participants were categorized into two groups based on their diabetes status: stroke patients with coexisting diabetes mellitus and stroke patients without diabetes mellitus. The estimated minimum sample size was 318 based on the standard formula for calculating differences in two proportions with a 95% confidence interval and a power of 95%.

Consecutive patients who fulfilled the inclusion criteria were enrolled into the study and their baseline demographic data such as age, sex, and marital and employment status were documented using a questionnaire. Medical history of diabetes, hypertension, and other comorbidities and complications during admission were also documented.

### 2.1. Physical Examination

Severity of neurological impairment was assessed within 24 hours of admission using the National Institutes of Health Stroke Scale (NIHSS) [16]. The neurological severity of the stroke was classified as minor (1-4), moderate (5-15), moderately-severe (16-20), and severe (21-42) with a score of zero indicating no stroke symptoms [16, 17]. Blood pressure and temperature measurements were taken and the swallowing assessment was performed on presentation for each participant.

### 2.2. Laboratory and Radiological Investigations

Fasting plasma glucose, fasting lipids, serum uric acid, electrolytes, urea, creatinine, and estimated glomerular filtration rate (eGFR) were evaluated within 24 hours of presentation for all participants. Glycated haemoglobin (HbA1c) was performed for all participants with elevated plasma glucose or diabetes. From the radiological imaging stroke was categorized broadly into ischaemic, haemorrhagic stroke and subarachnoid haemorrhage.

### 2.3. Definitions

Ischaemic stroke was defined as an episode of neurological dysfunction caused by focal cerebral infarction, haemorrhagic stroke was defined as rapidly developing clinical signs of neurological dysfunction attributable to a focal collection of blood within the brain parenchyma or ventricular system that is not caused by trauma, and subarachnoid haemorrhage was defined as bleeding into the subarachnoid space [18]. Diagnosis of diabetes was made in participants with history of diabetes or those treated with insulin and/or oral antidiabetic drugs. Patients not known to have diabetes but with repeated fasting plasma glucose ≥ 7.0mmol/L or random plasma glucose ≥11.1mmol/L or glycated haemoglobin ≥6.5% were also diagnosed with diabetes [19]. A good glycaemic control was defined as HbA1c less than 7% [19]. Hypertension was diagnosed based on a history of hypertension or treatment with antihypertensive medications or blood pressure ≥ 140/90mmHg [20] 24 hours after onset of stroke.

### 2.4. Stroke Outcome Assessment

The modified Rankin Scale (mRS) was used to measure the functional outcome [21]. It required an interview with the patient or caregivers and is scored on a hierarchical ordinal scale from 0 to 6, with 0 indicating no symptoms and 6 indicating death [21, 22]. Good and poor outcomes were defined by mRS score of 0 to 2 and 3 to 6, respectively [21]. We assessed all participants at 1, 3, and 6 months after stroke onset to determine stroke outcome. Patients who were unable to attend the clinic for the post-discharge assessment were contacted by telephone to determine the functional status or mortality using the mRS [23]. Post-stroke survival at the end of the study period was also assessed.

### 2.5. Ethical Issues

Ethical approval (MS-Et/M.2-P4.3 – 2014-2015) was obtained from the Ethical and Protocol Review Committee of the College of Health Sciences, University of Ghana. Participants or their next of kin (where necessary) gave informed consent before enrolment. All participants received appropriate multidisciplinary stroke care.

### 2.6. Statistical Analysis

Data was analyzed using the R-statistics. Demographic and clinical characteristics of stroke patients with and without DM were compared using the chi-squared and t-test for categorical and continuous variables, respectively. The functional outcomes at 1, 3, and 6 months after stroke based on diabetes status and stroke subtype were compared using chi-squared test. The pattern of survival in stroke patients with and without diabetes was assessed with Kaplan-Meier estimates of survival probability. To determine any statistically significant difference in survival between the 2 groups at the conventional 95% level, we employed the log-rank or Mantel-Haenszel test. Factors associated with functional outcome and survival were analyzed using univariate logistic regression. Significant risk factors were subsequently selected for multivariate logistic regression analysis to identify the baseline demographic and clinical variables most strongly related to each outcome after adjusting for potential confounders. Level of significance was set at p < 0.05.

## 3. Results

### 3.1. Demographic and Clinical Characteristics

In all, a total of 326 participants were involved, comprising 190 (58.3%) males and 136 (41.7%) females with a male: female ratio of 1.4:1. Age range was 28 to 92 years, with a mean age of 59.94±14.85 years. A total of 105 (32.20%) participants had diabetes, 62 (59.05%) of them had previously been diagnosed, and 43 (40.95%) were newly diagnosed with diabetes during their hospitalization. Among stroke patients with diabetes 54.29% (57) were female and 45.71% (48) were male with a male: female ratio of 1:1.2 (p= 0.002). The mean ages of 61.48±13.24 years and 57.63±14.67 years were obtained for stroke patients with and without DM, respectively (p= 0.018).

Overall 73.93% (241) of all stroke patients had a history of hypertension. At presentation 80.9% of patients in both groups were found to have elevated blood pressure. Significantly more DM patients had ischaemic stroke (71, 67.62%) compared with haemorrhagic stroke (29, 27.63%) p=0.004 with more than a 2-fold chance of having diabetes among those with ischaemic stroke compared with those with haemorrhagic stroke (OR=2.33, 95%CI= 1.41-3.93). Baseline demographic and clinical data are shown in Table 1. At admission, 35.24% (37) of DM patients and 28.51% (63) of patients without DM had moderately-severe to severe neurological impairment using the NIHSS (p=0.22). The mean fasting and random plasma glucose at admission were significantly higher for DM patients (10.43 ±5.33 and 8.29± 0.30) than for those without DM (6.84±2.16 and 5.91± 0.11) (p value< 0.001). Fifty-nine (56.19%) DM patients had glycated haemoglobin level equal to or greater than 7%.

### 3.2. Functional Outcome and Survival

Functional outcome among patients with and without DM is as shown in Table 2. One month after stroke onset 79.05% (83) of DM patients and 70.14% (155) of patients without DM had poor functional outcome (p= 0.09). At six months after stroke, 73.33% (77) of DM patients had a poor outcome compared with 61.99% (137) of those without DM based on the mRS (p=0.07).

**Figure 1:**
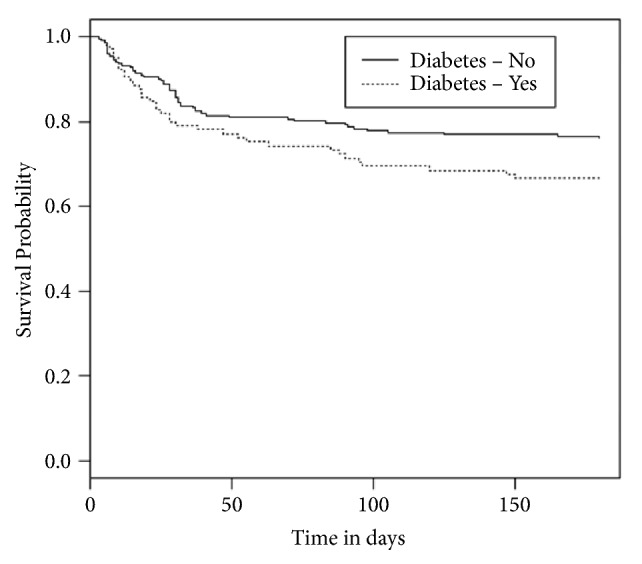
Kaplan-Meier survival curves for stroke patients with and without diabetes.

Mortality at 6 months after stroke among DM patients was 34.3% (36) whereas among patients without DM it was 26.9% (55) (p=0.10).  Figure 1 illustrates the pattern of survival among stroke patients with and without DM using Kaplan-Meier survival curves. Patients with DM were found to have a higher risk of mortality than their counterparts without DM. Employing the log-rank or Mantel-Haenszel test, the difference in survival between patient with and without DM was however not statistically significant (p-value=0.075).

Analyzing outcome data for ischaemic and haemorrhagic stroke separately, higher proportion of DM patients had poor outcome compared with those without DM (p>0.05) for both stroke subtypes. Mortality for each stroke subtype is as shown in Table 2. The 6-month mortality among ischaemic stroke patients was significantly higher for patients with DM compared with those without DM with the odds of mortality being two times that for those without DM (OR 2.037; 95%CI: 1.058-3.923; p=0.03).

### 3.3. Determinants of Outcome and Mortality

Univariate logistic regression analysis showed that age [OR1.04(1.03-1.06)], female gender [OR1.87(1.16-3.04)], unemployed status [OR3.87(1.87-8.84)], diagnosis of diabetes [OR1.66(1.01-2.81)], elevated temperature [OR1.45(1.10-1.94)], speech abnormalities [OR2.32(1.46-3.73)], high fasting plasma glucose on admission [OR1.15(1.03-1.29)], and diagnosis of pneumonia [OR5.54(2.85-11.89)] were associated with poor functional outcome among all study participants at the end of study period. Factors associated with functional outcome for patients with and without diabetes separately at 1 month, 3 months, and 6 months are as shown in Tables 3–5.

After a multivariate logistic regression analysis poor functional outcome was independently associated with older age (AOR=1.04, CI=1.01-1.06), female gender (AOR =1.85,CI=1.06-3.29), speech abnormalities (AOR=2.02,CI=1.20-3.46), and pneumonia (AOR=4.71,CI=2.30-10.53) among all stroke patients at the end of study. Among stroke patients with DM, determinants of poor functional outcome were older age, female gender, and diagnosis of pneumonia whereas for stroke patients without DM unemployed status, speech abnormalities, and pneumonia were the determinants of poor outcome (Tables 3–5).

Factors associated with low survival among all stroke patients were unemployed status [HR1.87(1.12-3.11)], high fasting plasma glucose [HR1.16(1.09-1.24)], elevated temperature [HR1.62(1.28-2.06)], and diagnosis of pneumonia [HR2.72(1.78-4.14)]. Predictors of mortality were high fasting plasma glucose [HR1.15(1.07-1.23)], elevated temperature [HR1.41(1.11-1.79)], and diagnosis of pneumonia [HR2.25(1.44-3.50)] among all stroke patients. Predictors of mortality for stroke patients with and without diabetes are as shown in Table 6.

## 4. Discussion

This study investigated the functional outcome and its associated factors among stroke patients with and without diabetes in Ghana. Diabetes was diagnosed in 32.2% of stroke patients and this is comparable with prevalence ranges reported from various parts of the world [7, 9, 24–26].Pathophysiological mechanisms including endothelial dysfunction, early arterial stiffness, and systemic inflammation contribute to the increased stroke occurrence among patients with diabetes [27].

Overall, more males were admitted with stroke compared with females in this study; however among patients with DM the proportion of females were significantly higher compared with patients without DM. Similar findings have previously been reported [24]. Females with DM have been shown to have a higher risk of stroke (3-6.5-fold) compared to their male counterparts (2-3-fold) [28, 29]. The effect of oestrogen and androgens on cardiovascular function have been suggested as one of the possible explanations for this difference [30]. The mean age of stroke patients with diabetes was significantly higher than those without diabetes; this is consistent with the findings of other studies [24, 29, 31].

Majority (73.93%) of stroke patients with and without DM had hypertension which is in keeping with results from other studies [24, 29, 31]. Generally, hypertension is more common among patients with DM with its attendant increased risk of cardiovascular complications including stroke [32]. Ischaemic stroke has been reported to be more prevalent among DM patients compared with haemorrhagic stroke and results from our study corroborate this finding [7, 31, 33, 34]. In our study the odds of having diabetes among patients with ischaemic stroke were more than two times (OR=2.33, 95% CI= 1.41-3.93) that among patients with haemorrhagic stroke.

In the current study a higher proportion of stroke patients with DM had poor functional outcome (at 1, 3, and 6 months) and reduced survival compared with their counterparts without DM; however the differences were not statistically significant. This may partly be because participants with both ischaemic and haemorrhagic strokes were included in this study whereas previous studies were mostly among patients with ischaemic stroke only. Secondly participants in this study received stroke unit care which has been associated with improved outcomes [35].

Diabetes patients with stroke have been reported to have significantly poorer functional outcome at 3 and 6 months in other studies [7, 24]. It has been suggested that patients with diabetes recover more slowly from stroke compared with those without diabetes [7, 36, 37]. Various reasons have been suggested for this finding and this includes patients with diabetes having more comorbidities, more pre-stroke disability, more lacunar infarcts, more motor problems, and diabetic neuropathy [7]. Pathophysiologically there is increasing evidence that DM directly affects the Central Nervous System and limits the brain's innate capacity for repair and rewiring which are critical for successful stroke recovery [38, 39].

Studies from other populations have suggested that DM is associated with a higher mortality from stroke [6–8] whereas other reports did not corroborate this finding [12, 13]. Mortality at 3 and 6 months was found to be significantly higher among stroke patients with diabetes compared with those without diabetes in China [24]. In contrast in another study, case fatality at 3 months was found to be comparable for patients with and without DM [7]. Possible explanations for these findings could be the differences in study design, characteristics of study population including genetic make-up, cultural differences, and pre-stroke level of glycaemic control. Additionally, variations in the socioeconomic background and its resultant impact on availability of recommended stroke management interventions may also play a role.

Diabetes has been shown to be an independent risk factor for death [24] or poor functional outcome at 3 and 6 months among stroke patients in other studies [7, 24]. In contrast, in this study although diabetes was associated with poor functional outcome it was not an independent risk factor after multivariate analysis. Considering stroke patients with DM separately, determinants of poor functional outcome were older age and female gender whereas those for stroke patients without DM were unemployed status and speech abnormalities. Similarly, female gender and older age have been shown to be predictors of poor stroke outcome among DM patients in a multicenter European study [7]. Studies suggest that poor functional outcome among women may be attributable to the incidence of stroke at an older age among women, presence of more comorbidities, and occurrence of more severe stroke among women than men [40, 41]. Other predictive factors for poor outcome among diabetes patients include pre-stroke Rankin Scale score 2 to 5, urinary incontinence, and coma [7].

Predictors of reduced survival in this study were high fasting plasma glucose and elevated temperature on presentation. Admission hyperglycemia has also been shown elsewhere to predict a worse functional outcome and mortality after ischaemic stroke [42, 43]. In the UKPDS trial a 1% rise in HBA1c was associated with a 37% increase in stroke case fatality [44]. It has been suggested that glucose may exert a deleterious effect on ischaemic brain through the activation of several cellular mechanisms [45]. It has been proposed that baseline hyperglycemia may also represent an acute stress response from activation of the hypothalamic-pituitary-adrenal axis causing an increase in cortisol and catecholamines and may be indicative of underlying stroke severity. Furthermore, it is thought that hyperglycaemia may be a result of injury or irritation of brain areas involved in glucose regulation [46].

A diagnosis of pneumonia in this study was found to be an independent risk factor for both poor functional outcome and reduced survival for patients with and without diabetes and this is similar to previous reports [28, 47]. Pneumonia is diagnosed in up to 30% of stroke patients and is reported to be associated with the highest attributable mortality after a stroke [48, 49]. Predictors of post-stroke pneumonia include age greater than 65 years, speech abnormalities, modified Rankin Scale greater than 4, abbreviated mental test score less than 8, and a failed swallowing test [48]. The use of a multidisciplinary team approach for the early detection of stroke patients at high risk of post-stroke pneumonia and the implementation of appropriate preventive and therapeutic interventions has been shown to improve outcomes [50].

In Ghana noncommunicable diseases including stroke and diabetes are increasingly becoming major public health burden [51]. Highlighting the risk factors associated with adverse stroke outcome is expected to facilitate the early recognition of at-risk patients and the institution of appropriate interventions to improve outcomes. This was hospital-based study and therefore the results may not be generalizable to Ghanaian population; however, the prospective design is a methodological strength of this study.

## 5. Conclusion

We found that there was the tendency for stroke patients with diabetes to have a poor functional outcome and reduced survival compared with their counterparts without diabetes. Older age, female gender, elevated temperature, high fasting plasma glucose, and pneumonia were determinants of adverse outcome in stroke patients with diabetes whereas speech abnormalities, unemployed status, and pneumonia were the determinants for those without diabetes. The early identification of these factors will facilitate appropriate management to eliminate or minimize adverse effects and improve outcomes among Ghanaian stroke patients especially those with diabetes.

## Figures and Tables

**Table 1:** Diabetes status versus baseline demographic and clinical characteristics among stroke patients admitted at Korle Bu Teaching Hospital.

	**Had Diabetes**		**P-value**
	**No(N/**%**)**	**Yes(N/**%**)**	

**Gender**			
Male	142(64.25)	48(45.71)	
Female	79(35.75)	57(54.29)	0.002
**Mean Age (SD) years**	57.63(0.99)	61.48(1.29)	0.018
**PMHx Hypertension **			
Yes	155(70.14)	86(81.9)	0.0237
No	66(29.86)	19(18.1)	
**Swallowing test**			
Pass	161(72.85)	70(68.57)	0.472
Failed	60(27.15)	35(31.43)	
**Had speech abnormalities**			
No	112(50.68)	48(45.71)	0.40
Yes	109(49.32)	57(54.29)	
**Mean systolic BP (SD) mmHg**	152.29(2.22)	156.74(3.43)	0.266
**Mean diastolic BP (SD) mmHg **	93.03(1.36)	91.47(2.30)	0.536
**Temperature**			
Normal	48(21.72)	20(19.05)	0.683
Fever	173(78.28)	85(80.95)	
**Haemorrhagic Stroke**	103(46.61)	29(27.62)	0.004
**Ischaemic Stroke**	108(48.87)	71(67.62)	
**Other **	10(4.52)	5(4.76)	
**Pneumonia **			
Yes	59 (26.7)	26 (24.76)	0.710
No	162(73.3)	79 (75.24)	
**eGFR **			
>60ml/hr/1.73m2	163(78.52)	75(71.43)	0.758
<60ml/hr/1.73m2	58(21.48)	30(28.57)	
**Total Cholesterol (mmol/L)**	5.48(0.11)	5.10(0.17)	0.065
**LDLc (mmol/L) **	3.55(0.10)	3.24(0.15)	0.094
**HDLc (mmol/L) **	1.60(0.14)	1.38(0.06)	0.149
**Triglycerides (mmol/L)**	1.97(0.53)	1.30(0.08)	0.208
**Uric Acid (umol/L)**	345.93(11.99)	345.78(15.24)	0.994
**Random Blood Glucose (mmol/L)**	6.84(0.15)	10.43(0.52)	<0.001
**Fasting Plasma Glucose (mmol/L)**	5.91(0.11)	8.29(0.30)	<0.001

BP: blood pressure, eGFR: estimated glomerular filtration rate, LDLc: low density lipoprotein cholesterol, and HDLc: high density lipoprotein cholesterol.

**Table 2:** Severity of neurological impairment and stroke outcome versus diabetes status.

	**No Diabetes N(**%**)**	**Diabetes N(**%**)**	**p-value**

**Baseline NIHSS **			0.22
Severe	63(28.51)	37 (35.24)	
Not severe	158 (71.49)	68 (64.76)	

**mRS at 1 month**			0.09
Poor	155 (70.14)	83 (79.05)	
Good	66 (29.86)	22 (20.95)	

**mRS at 3 months**			0.09
Poor	144 (65.16)	79 (75.24)	
Good	77 (34.84)	26 (24.76)	

**mRS at 6 months**			0.07
Poor	137 (61.99)	77(73.33)	
Good	84 (38.01)	28(26.67)	

**Mortality at 1 month (Total)**	35(15.8)	22(20.95)	0.26
**Ischaemic stroke**	16(14.8)	15(21.1)	0.27
**Haemorrhagic stroke**	18(17.6)	6(20.0)	0.69

**Mortality at 3 months (Total)**	49(21.7)	31(29.5)	0.12
**Ischaemic stroke**	21(19.44)	22(31)	0.08
**Haemorrhagic stroke**	26(25.2)	8(27.6)	0.80

**Mortality at 6 months (Total)**	55(26.9)	36 (34.3)	0.10
**Ischaemic stroke**	25(23.1)	27(38)	0.03
**Haemorrhagic stroke**	29(28.2)	8(27.6)	0.95

**Table 3:** Factors associated with functional outcome at 1 month for stroke patients with and without diabetes on univariate and multivariate logistic regression analysis.

	**Univariate**		**Multivariate**	

	**DM**	**No DM**	**DM**	**No DM**
**Variables**	AOR(95% CI)	AOR(95% CI)	AOR(95% CI)	AOR(95% CI)

**Age**	1.08(1.04-1.13)*∗∗∗*	1.02(1.00-1.05)*∗*	1.09(1.03-1.16)*∗∗*	1.01(0.98-1.04)
**Employment status**				
Employed	1.00(Reference)	1.00(Reference)	1.00(Reference)	1.00(Reference)
Unemployed	2.46(0.71-11.54)	5.92(1.99-25.53)*∗∗*	0.82(0.17-4.49)	4.19(1.24-19.50)*∗*
Retired	1.98(1.05-16.38)*∗*	1.67(0.81-3.62)	0.63(0.10-4.11)	1.08(0.42-2.84)
**FPG**	-	1.26(1.01-1.61)*∗*	-	1.14(0.89-1.50)
**Temperature**	-	1.65(1.13-2.51)*∗*	-	1.38(0.89-2.19)
**Pneumonia**				
No	-	1.00(Reference)	-	1.00(Reference)
Yes	-	5.20(2.26-14.15)*∗∗∗*	-	3.92(1.64-10.92)*∗∗*

*∗*Significant at 5% level, *∗∗*significant at 1% level, and *∗∗∗*significant at 0.1% level.

**Table 4:** Factors associated with functional outcome at 3 months for stroke patients with and without diabetes on univariate and multivariate logistic regression analysis.

	**Univariate**		**Multivariate**	

	**DM**	**No DM**	**DM**	**No DM**
**Variables**	AOR(95% CI)	AOR(95% CI)	AOR(95% CI)	AOR(95% CI)

**Age**	1.09(1.04-1.14)*∗∗∗*	1.02(1.01-1.05)*∗∗*	1.12(1.05-1.21)*∗∗∗*	1.02(0.99-1.05)
**Gender**				
Male	1.00(reference)	-	1.00 (Reference)	-
Female	2.92(1.18-7.65)*∗*	-	3.74(1.26-12.65)*∗*	-
**Employment status**				
Employed	1.00(Reference)	1.00(Reference)	1.00(Reference)	1.00(Reference)
Unemployed	3.17(0.92-14,73)	4.10(1.62-12.62)*∗∗*	0.47(0.08-2.93)	2.45(0.83-8.33)
Retired	3.30(1.08-12.43)*∗*	1.67(0.83-3.47)	0.28(0.04-1.87)	0.79(0.31-1.98)
**Temperature**	-	1.63(1.13-2.42)*∗*	-	1.49(0.98-2.30)
**Speech abnormalities**				
No	-	1.00(Reference)	-	1.00(Reference)
Yes	-	1.75(1.00-3.09)*∗*	-	1.92(1.04-3.59)*∗*
**Pneumonia**				
No	1.00(Reference)	1.00(Reference)	1.00(Reference)	1.00(Reference)
Yes	11.57(2.25-212.49)*∗*	4.73(2.21-11.35)*∗∗∗*	10.46(1.75-204.60)*∗*	3.85(1.72-9.57)*∗∗*

*∗*Significant at 5% level, *∗∗*significant at 1% level, and *∗∗∗*significant at 0.1% level.

**Table 5:** Factors associated with functional outcome at 6 months for stroke patients with and without diabetes on univariate and multivariate logistic regression analysis.

	**Univariate**		**Multivariate**	

	**DM**	**No DM**	**DM**	**No DM**

**Variables**	AOR(95% CI)	AOR(95% CI)	AOR(95% CI)	AOR(95% CI)
**Age**	1.08(1.04-1.13)*∗∗∗*	1.03(1.01-1.05)*∗∗*	1.07(1.03-1.12)*∗∗*	1.02(0.99-1.05)
**Employment status**				
Employed	-	1.00(Reference)	-	1.00(Reference)
Unemployed	-	3.93(1.63-11.04)*∗∗*	-	2.17(0.75-6.89)
Retired	-	1.98(0.99-4.13)	-	0.76(0.30-1.96)
**Temperature**	-	1.54(1.08-2.24)*∗*	-	1.32(0.87-2.05)
**Speech abnormalities**				
No	1.00(Reference)	1.00(Reference)	1.00(Reference)	1.00(Reference)
Yes	3.62(1.48-9.44)*∗∗*	1.93 (1.11-3.37)*∗*	2.09(0.74-6.04)	1.99(1.08-3.73)*∗*
**FPG**	-	1.25(1.03-1.57)*∗*	-	1.15(0.90-1.49)
**Pneumonia **				
No	1.00(Reference)	1.00(Reference)	1.00(Reference)	1.00(Reference)
Yes	13.24(2.58-242.79)*∗*	4.79(2.30-11.00)*∗∗∗*	11.32(1.93-220.05)*∗*	4.05(1.83-9.77)*∗∗∗*

*∗*Significant at 5% level, *∗∗*significant at 1% level, and *∗∗∗*significant at 0.1% level.

**Table 6:** Determinants of survival of stroke patients with and without diabetes.

**Variables**	**Diabetes**		**No diabetes**	
	Coefficient (b_i_)	HR (95% CI)	Coefficient (b_i_)	HR (95%CI)

**Pneumonia**				
No	0.00	1.00 (Reference)	0.00	1.00 (Reference)
Yes	0.81	2.24 (1.05, 4.82)*∗*	0.72	2.05 (1.13, 3.74)*∗*
**Temperature **	0.37	1.44 (1.01, 2.07)*∗*	0.41	1.51 (1.04, 2.19)*∗*
**FPG **	0.13	1.14 (1.02, 1.28)*∗*	0.22	1.25 (1.1,1.42)*∗∗∗*
**Employment status**				
Employed	0.00	1.00 (Reference)	0.00	1.00 (Reference)
Unemployed	0.67	1.96 (0.74, 5.16)	0.35	1.43 (0.72, 2.81)
Retired	0.81	2.24 (0.86, 5.85)	-0.600	0.55 (0.24, 1.22)
**Hypertension**				
No	0.00	1.00 (Reference)	0.00	1.00 (Reference)
Yes	0.45	1.56 (0.56, 4.36)	-0.270	0.76 (0.4, 1.46)
**Stroke subtypes**				
Haemorrhagic	0.00	1.00 (Reference)	0.00	1.00 (Reference)
Ischaemic	-0.17	0.84 (0.31, 2.26)	-0.120	0.88 (0.5, 1.56)
Others	-0.47	0.62 (0.08, 5.13)	-0.970	0.38 (0.05, 2.82)

**A1c status **				
>7%	0.00	1.00 (Reference)		
≤7%	0.16	1.17 (0.55, 2.5)		

CI: confidence interval, HR: hazard ratio, FPG: fasting plasma glucose, and A1c: glycated haemoglobin; *∗*significant at 5% level, *∗∗*significant at 1% level, and *∗∗∗*significant at 0.1% level.

## Data Availability

The data used to support the findings of this study are available from the corresponding author upon request.
